# Rab3a-Bound CD63 Is Degraded and Rab3a-Free CD63 Is Incorporated into HIV-1 Particles

**DOI:** 10.3389/fmicb.2017.01653

**Published:** 2017-08-29

**Authors:** Yoshinao Kubo, Hiroshi Masumoto, Mai Izumida, Katsura Kakoki, Hideki Hayashi, Toshifumi Matsuyama

**Affiliations:** ^1^Department of Molecular Microbiology and Immunology, Graduate School of Biomedical Sciences, Nagasaki University Nagasaki, Japan; ^2^Program for Nurturing Global Leaders in Tropical and Emerging Communicable Diseases, Graduate School of Biomedical Sciences, Nagasaki University Nagasaki, Japan; ^3^Biomedical Research Support Center, Nagasaki University School of Medicine Nagasaki, Japan; ^4^Department of Clinical Medicine, Institute of Tropical Medicine, Nagasaki University Nagasaki, Japan; ^5^Department of Urology, Graduate School of Biomedical Sciences, Nagasaki University Nagasaki, Japan; ^6^Medical University Research Administrator, Nagasaki University School of Medicine Nagasaki, Japan

**Keywords:** CD63, HIV-1, Rab3a, virion formation, endosome/lysosome

## Abstract

CD63, a member of the tetraspanin family, is involved in virion production by human immunodeficiency virus type 1 (HIV-1), but its mechanism is unknown. In this study, we showed that a small GTP-binding protein, Rab3a, interacts with CD63. When Rab3a was exogenously expressed, the amounts of CD63 decreased in cells. The Rab3a-mediated reduction of CD63 was suppressed by lysosomal and proteasomal inhibitors. The amount of CD63 was increased by reducing the endogenous Rab3a level using a specific shRNA. These results indicate that Rab3a binds to CD63 to induce the degradation of CD63. Rab3a is thought to be involved in exocytosis, but we found that another function of Rab3a affects the fate of CD63 in lysosomes. CD63 interacted with Rab3a and was incorporated into HIV-1 particles. However, Rab3a was not detected in HIV-1 virions, thereby indicating that Rab3a-free CD63, but not Rab3a-bound CD63, is incorporated into HIV-1 particles. Overexpression or silencing of Rab3a moderately reduced HIV-1 virion formation. Overexpression of Rab3a decreased CD63 levels, but did not affect the incorporation of CD63 into HIV-1 particles. This study showed that Rab3a binds to CD63 to induce the degradation of CD63, and only Rab3a-free CD63 is incorporated into HIV-1 particles.

## Introduction

The main targets of human immunodeficiency virus type 1 (HIV-1) are CD4-positive T lymphocytes, but it is also important to understand the mechanism of HIV-1 virion production in fibroblast cell lines. HIV-1 is used frequently as a vesicle to transfer a gene of interest. The HIV-1 vector is constructed by transfecting 293T or COS7 fibroblast cells expressing the T antigen of simian virus 40 (SV40), which induces DNA replication of SV40 replication origin-containing plasmids. Understanding the mechanism of HIV-1 virion production in these cell lines would contribute to the construction of highly efficient or cell type-specific HIV-1 vectors.

Some members of the tetraspanin family are associated with HIV-1 replication. Tetraspanin family members including CD9, CD63, CD81, and CD82 are membrane-spanning proteins with four transmembrane domains, and these proteins form tetraspanin-enriched microdomains (TEMs) in plasma membranes. Tetraspanins and TEMs participate in many physiological and pathological events (Andreu and Yanez-Mo, 2014; Mazzocca et al., 2014; Jiang et al., 2015).

The tetraspanin proteins expressed by host cells affect the early steps of the HIV-1 life cycle. CD63 silencing in host cells reportedly inhibits HIV-1 Env-mediated entry (Li et al., 2011, 2014). It has been shown that CD63 controls the trafficking of CXCR4, an HIV-1 receptor, and modulates the cellular susceptibility to CXCR4-tropic HIV-1 infection (Yoshida et al., 2008).

Tetraspanins are also involved in the late steps of the HIV-1 replication cycle, where the HIV-1 virions are formed in TEMs (Jolly and Sattentau, 2007), and the tetraspanin proteins are incorporated into HIV-1 virions (Chertova et al., 2006). CD63, a member of the tetraspanin family, is also incorporated into HIV-1 particles, but CD63 is specifically localized to the late endosomes/lysosomes, whereas the HIV-1 virion is thought to be formed in the cell surface plasma membrane (Votteler and Sundquist, 2013), thereby suggesting that HIV-1 employs a positive mechanism for preferentially incorporating CD63 into virions. The knockdown of CD63 (Chen et al., 2008; Fu et al., 2015) or CD81 (Grigorov et al., 2009) reportedly inhibits HIV-1 virion production, which indicates that tetraspanins are required for HIV-1 virion production. By contrast, it has been reported that HIV-1 infectivity is attenuated by the incorporation of tetraspanin proteins into HIV-1 particles, although exogenous expression of the wild type (WT) CD63 did not affect the infectivity of the VSV-pseudotyped HIV-1 vector (Sato et al., 2008; Kubo et al., 2016). Interestingly, it has been reported that the expression of tetraspanins is downregulated by HIV-1 Vpu to enhance infectivity (Haller et al., 2014; Lambele et al., 2015), which suggests that tetraspanins are negative regulators of HIV-1 replication. At present, it is thought that tetraspanin proteins have both beneficial roles in virion formation and detrimental effects on the infectivity of viral particles (Lambele et al., 2015). However, the molecular role of tetraspanins in HIV-1 virion formation still needs to be elucidated.

Recently, we reported that gamma-interferon-inducible lysosomal thiol reductase (GILT) inhibits HIV-1 virion production by digesting the disulfide bonds in CD63 (Kubo et al., 2016). CD63 is localized specifically to endosomes and lysosomes in the same manner as GILT, and it has conserved cysteine residues that form disulfide bonds (Hemler, 2008). We found that a CD63 mutant containing amino acid substitutions of the conserved cysteine residues with serine inhibited HIV-1 virion production.

In this study, to understand the mechanism that regulates CD63 during HIV-1 virion production, we aimed to identify the cellular proteins that interact with CD63. We found that Rab3a is a CD63-interacting protein. Rab3a is a small GTP-binding protein and it is associated with exocytosis (Branham et al., 2009). In addition, the involvement of Rab3a with lysosomal functions has been reported recently (Encarnacao et al., 2016). Thus, we analyzed the effects of Rab3a on CD63 expression and HIV-1 virion formation in this study.

## Results

### CD63 Is Required for HIV-1 Virion Production

**FIGURE 1:**
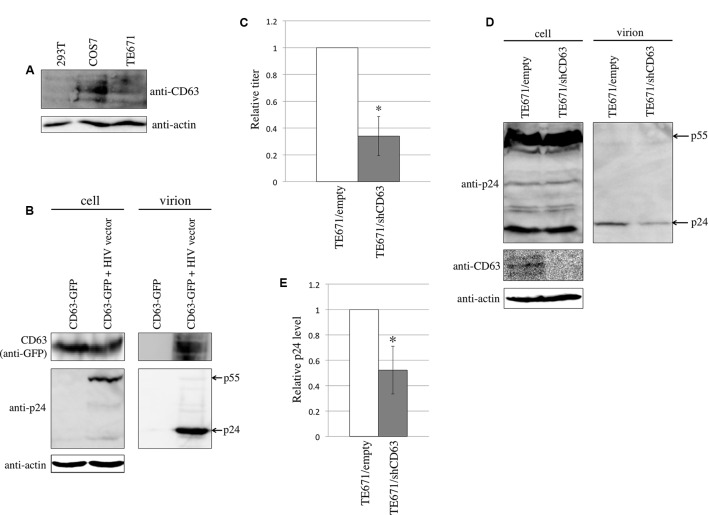
CD63 was incorporated into HIV-1 particles and was required for HIV-1 virion production. **(A)** CD63 expression in 293T, COS7, and TE671 was analyzed by western blotting using anti-CD63 antibody. **(B)** 293T cells were transfected using CD63 WT-GFP together with pcDNA3.1 or HIV-1 vector construction plasmids. Cell lysates and virion pellets were analyzed by western blotting. **(C)** TE671 cells transduced by empty or shCD63-encoding lentivirus vector were transfected using the VSV-pseudotyped HIV-1 vector construction plasmids. Culture supernatants from the transfected cells were used to inoculate TE671 cells. Transduction titers from the empty vector-transduced cells were set to 1 and the relative values ± SD are indicated. Asterisk indicates significant differences. **(D)** Cell lysates and virion pellets prepared from the transfected TE671 cells were analyzed by western blotting. **(E)** The p24 levels in cell lysates were normalized against the actin levels, and the p24 levels in virion pellets were normalized against the normalized p24 levels. The normalized p24 levels in the virion pellets from TE671 cells transduced by empty vector cells were set to 1 and the relative values ± SD are indicated. Asterisk indicates significant differences.

To analyze CD63 expression in 293T, COS7, and TE671 cells that were used in this study, western blotting was performed using an anti-CD63 antibody. CD63 was detected in COS7 and TE671 cells, but 293T cells express CD63 at a relatively lower level (**Figure 1A**).

To assess whether CD63 is incorporated into HIV-1 particles, 293T cells were transfected using a C-terminally GFP-tagged CD63 (CD63 WT-GTP) expression plasmid with or without the HIV-1 vector construction plasmids. The HIV-1 particles were collected by centrifugation of the culture supernatants from the transfected cells through 20% sucrose. CD63 WT-GFP protein was detected in the virion-containing pellets, only when 293T cells were transfected with the HIV-1 vector construction plasmids (**Figure 1B**). This result showed that CD63 is incorporated into HIV-1 particles, as already reported (Chertova et al., 2006).

To examine whether CD63 is required for HIV-1 virion production, TE671 cells stably expressing an shRNA against CD63 (shCD63) were constructed using a lentivirus vector. The CD63-silenced TE671 cells were transfected using the VSV-pseudotyped HIV-1 vector construction plasmids, and the culture supernatants were inoculated into TE671 cells. Sato et al. (2008) have previously reported that CD63 incorporated into HIV-1 particles inhibits HIV-1 envelope protein-mediated infection, but not VSV-G-mediated infection. To measure the impact of CD63 only on HIV-1 virion production, we used VSV-pseudotyped HIV-1 vector. The CD63 silencing reduced transduction titers (**Figure 1C**). CD63 levels in the shCD63-expressing cells were indeed lower than those in the empty vector-transduced cells, confirming the inhibition of CD63 expression by the shCD63 (**Figure 1D**). HIV-1 p24 levels in cell lysates were not changed, but those in virion pellets were reduced by the CD63 silencing (**Figures 1D,E**). These results suggested that CD63 is required for efficient HIV-1 virion production, as already reported (Chen et al., 2008; Fu et al., 2015).

### Isolation of CD63-Interacting Proteins

Previously, we reported that a CD63 mutant (TCS) containing amino acid substitutions at four of the six conserved cysteine residues with serine inhibited HIV-1 virion production (Kubo et al., 2016). Thus, cellular factors that bind to CD63 WT but not to CD63 TCS mutant may be associated with HIV-1 virion formation. To isolate CD63-binding proteins involved with HIV-1 virion formation, expression plasmids encoding the CD63 WT and TCS mutant C-terminally tagged with influenza virus hemagglutinin (HA) and 6-times histidine–asparagine (6× HN) were constructed (CD63 WT-HA-6× HN and CD63 TCS-HA-6× HN). COS7 cells were transfected using these expression plasmids and their cell lysates were applied to Ni columns. Because GILT inhibited HIV-1 virion production in COS7 cells but not in 293T cells (Kubo et al., 2016), we used COS7 cells to isolate CD63-binding proteins. The CD63-binding proteins were eluted using an imidazole-containing buffer and fractionated to obtain 0.5 ml of each. The fractions were then analyzed by western blotting using an anti-HA antibody to determine the fractions containing CD63. Fraction 7 of CD63 WT-HA-6× HN and fraction 5 of CD63 TCS-HA-6× HN contained the highest CD63 protein levels (**Supplementary Figure S1A**). The CD63-containing fractions were subjected to SDS-PAGE, followed by silver staining. An additional 25-kDa protein band was detected in the CD63 WT fraction, but not in the CD63 TCS fraction (**Supplementary Figure S1B**). The extra protein was identified as Rab3a using mass spectrometry.

### Rab3a Induced CD63 Degradation

Rab3a is a small GTP-binding protein, where the GTP- and GDP-binding forms of Rab3a are active and inactive, respectively. A Rab3a mutant containing an amino acid substitution of a threonine residue at position 36 with asparagine (T36N) cannot bind GTP and it functions as a constitutively inactive mutant (Burstein et al., 1992). Another Rab3a mutant containing an amino acid substitution of a glutamine residue at position 81 with leucine (Q81L) lacks a GTPase activity and functions as a constitutively active mutant (Star et al., 2005; Huang et al., 2011). We constructed plasmids expressing the C-terminally HA-tagged Rab3a WT, T36N, and Q81L (Rab3a WT-HA, T36N-HA, and Q81L-HA) to analyze the effects of the activation state of Rab3a.

**FIGURE 2:**
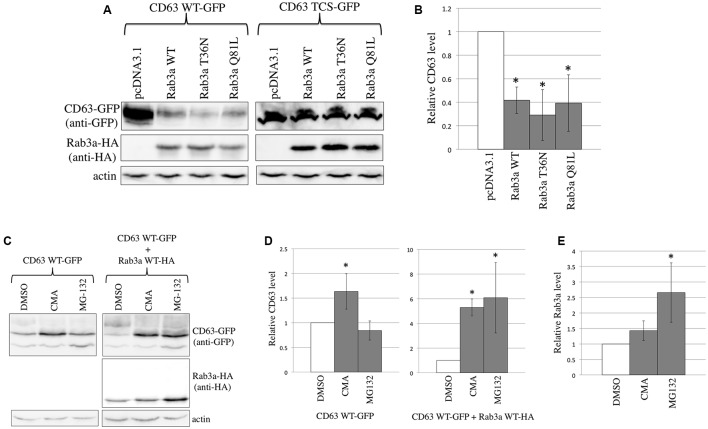
Rab3a induced the degradation of CD63. **(A)** 293T cells were transfected using CD63 WT-GFP or CD63 TCS-GFP together with pcDNA3.1, HA-tagged Rab3a WT, T36N, or Q81L. Cell lysates prepared from the transfected cells were analyzed by western blotting using anti-GFP, anti-HA, and anti-actin antibodies. **(B)** The CD63 levels were normalized against the actin levels. The normalized CD63 levels in the pcDNA3.1-transfected cells were set to 1 and the relative values ± SD are indicated. Asterisks indicate significant differences compared with the values in the pcDNA3.1-transfected cells. **(C)** 293T cells were transfected using CD63 WT-GFP together with pcDNA3.1 or Rab3a WT-HA. The transfected cells were treated with DMSO, CMA, or MG-132. Cell lysates from the treated cells were analyzed by western blotting using anti-GFP, anti-HA, and anti-actin antibodies. **(D)** The CD63 levels were normalized against the actin levels. The normalized CD63 levels in the DMSO-treated cells were set to 1 and the relative values ± SD are indicated. Asterisks indicate significant differences compared with the values in the DMSO-treated cells. **(E)** The Rab3a levels were normalized against the actin levels.

To confirm binding between CD63 and Rab3a, 293T cells were transfected using the expression plasmid of C-terminally GFP-tagged CD63 WT (CD63 WT-GFP) together with pcDNA3.1, Rab3a WT-HA, T36N-HA, or Q81L-HA. Surprisingly, we noted that the CD63 WT expression levels were decreased when Rab3a WT-HA, T36N-HA, or Q81L-HA was co-expressed (**Figures 2A,B**). By contrast, the expression levels of CD63 TCS-GFP were not affected by Rab3a expression, thereby supporting the result that CD63 TCS did not interact with Rab3a. These results suggest that Rab3a reduces the CD63 expression level independently of the Rab3a activation state.

**FIGURE 3:**
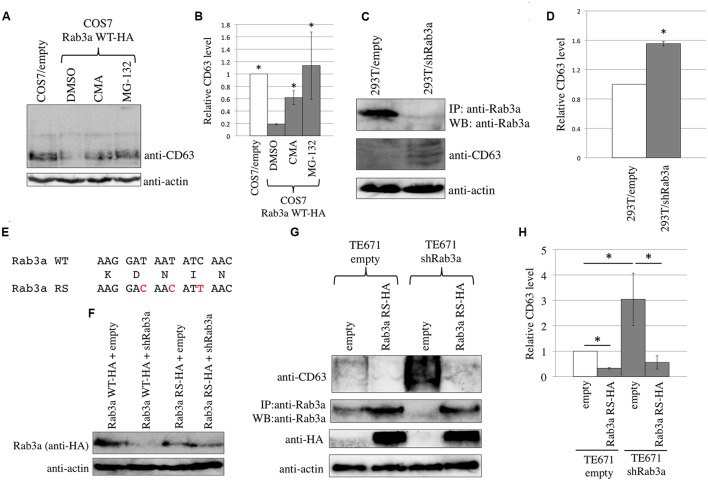
Endogenous Rab3a induced the degradation of endogenous CD63. **(A)** COS7 cells transduced using the empty or Rab3a WT-HA-encoding MLV vector were treated with DMSO, CMA, or MG-132. Cell lysates were analyzed by western blotting using anti-CD63 and anti-actin antibodies. **(B)** Endogenous CD63 levels were normalized against the actin levels. The normalized CD63 levels in the DMSO-treated, Rab3a WT-HA-expressing cells were set to 1 and the relative values ± SD are indicated. Asterisks indicate significant differences compared with the values in the DMSO-treated, Rab3a WT-HA-expressing cells. **(C)** 293T cells were transduced using the shRab3a-encoding lentivirus vector. Their cell lysates were immunoprecipitated with anti-Rab3a antibody. The precipitates were analyzed by western blotting using anti-Rab3a antibody. Cell lysates were also analyzed by western blotting using anti-CD63 and anti-actin antibodies. **(D)** The endogenous CD63 levels were normalized against the actin levels. The normalized CD63 levels in the empty vector-transduced cells were set to 1 and the relative values ± SD are indicated. Asterisk shows statistically significant differences to the values in the empty vector-transduced cells. **(E)** Nucleotide (upper and lower lines) and amino acid (middle line) sequences of the shRab3a target in Rab3a WT and Rab3a RS are indicated. Red characters indicate nucleotide substitutions in the Rab3a RS. **(F)** 293T cells were transfected using Rab3a WT-HA or Rab3a RS-HA (100 ng) together with empty or shRab3a-expressing plasmid (1 μg). Cell lysates from the transfected cells were analyzed by western blotting with anti-HA and anti-actin antibodies. **(G)** TE671/empty or TE671/shRab3a cells were inoculated with the Rab3a-RS-HA-encoding MLV vector. Cell lysates were analyzed by western blotting with anti-CD63, anti-HA, and anti-actin antibodies. Precipitates by anti-Rab3a antibody were analyzed by western blotting with the same antibody. **(H)** The endogenous CD63 levels were normalized against the actin levels. The normalized CD63 levels in the empty vector-transduced cells were set to 1 and the relative values ± SD are indicated. Asterisks show statistically significant differences between the two groups.

To determine whether Rab3a induces the degradation of CD63 in lysosomes or proteasomes, the transfected cells were treated with a lysosome inhibitor, concanamycin A (CMA), or a proteasome inhibitor, MG-132. When 293T cells were transfected using the CD63 WT-GFP expression plasmid alone, CMA treatment increased the amount of CD63 by 1.5 times, whereas treatment with MG-132 did not (**Figures 2C,D**). Thus, CD63 is constitutively degraded in the lysosomes, which is consistent with its specific localization to the lysosomes. Next, 293T cells were co-transfected using the CD63 WT-GFP and Rab3a WT-HA expression plasmids, and then treated with the inhibitors. Treatment with either CMA or MG-132 increased the amount of CD63 by six times (**Figures 2C,D**). In addition, the Rab3a level was increased by the treatment with MG-132 (**Figures 2C,E**). However, CD63 and Rab3a proteins with higher molecular sizes than expected were not detected (**Figures 2C**, **3A**), thereby showing that these proteins were not polyubiquitinated. These results suggest that Rab3a activates the lysosomal degradation of CD63, and that proteasomal degradation is indirectly associated with the Rab3a-mediated reduction of the CD63 level.

To determine whether Rab3a reduces the endogenous CD63 levels, COS7 cells that originally expressed CD63 at a relatively higher level were inoculated with a murine leukemia virus (MLV) vector encoding Rab3a WT-HA. COS7 cells were also inoculated with a MLV vector encoding Rab3a T36N-HA or Q81L-HA, and then selected by puromycin, but resistant colonies did not appear, which suggests that these mutant proteins induced cell death or stopped cell growth. In COS7 cells that stably expressed Rab3a WT-HA, the endogenous CD63 level was lower compared with that in cells transduced with an empty MLV vector (**Figures 3A,B**). Treatment of the Rab3a WT-HA-expressing cells with the lysosomal or proteasomal inhibitor increased the endogenous CD63 level. Thus, exogenous Rab3a expression induced the degradation of endogenous CD63.

To determine whether endogenous Rab3a reduced the endogenous CD63 levels, 293T cells that originally expressed CD63 at a relatively lower level were inoculated with a lentiviral vector expressing an shRNA against Rab3a mRNA (shRab3a). The Rab3a protein band was faint after direct western blotting of the cell lysates prepared from 293T cells, so the Rab3a protein was concentrated by immunoprecipitation using a rabbit anti-Rab3a antibody and the precipitates were then analyzed by western blotting using the anti-Rab3a antibody. An HRP-conjugated anti-native rabbit IgG antibody was used as the secondary antibody to avoid detecting denatured rabbit IgG present in the precipitates. The Rab3a levels were lower in the shRab3a-expressing 293T cells than the empty vector-transduced cells (**Figure 3C**), thereby confirming the shRab3a-mediated knockdown of Rab3a expression. The endogenous CD63 levels were increased in the Rab3a-silenced cells (**Figures 3C,D**). Thus, the endogenous Rab3a decreased the amount of endogenous CD63.

To examine whether the shRab3a elevates CD63 level by unexpected off-target effects, we constructed a plasmid expressing Rab3a WT-HA protein resistant to the shRab3a (Rab3a RS-HA). The shRab3a target sequence of the Rab3a WT-HA expression plasmid was substituted by a synonymous sequence (**Figure 3E**). When 293T cells were transfected using the expression plasmid of Rab3a WT-HA or Rab3a RS-HA (100 ng) together with the empty or shRab3a-encoding plasmid (1 μg), Rab3a WT-HA expression levels were decreased by the shRab3a, but not Rab3a RS-HA (**Figure 3F**). TE671 cells were transduced by the shRab3a-expressing lentivirus vector, and were selected with puromycin. The shRab3a-transduced TE671 cells were inoculated with the Rab3a RS-HA-expressing MLV vector. Because the shRab3a-transduced TE671 cells were originally resistant to puromycin, the cells inoculated with the MLV vector expressing the Rab3a RS-HA and puromycin-resistant genes were not treated with puromycin. When normal TE671 cells were inoculated with the Rab3a RS-HA expressing MLV vector, almost all cells survived by puromycin selection. Thus, high majority of the inoculated cells should express Rab3a RS-HA protein without puromycin selection. Cell lysates from the transduced cells were immunoprecipitated with the anti-Rab3a antibody, and the precipitates were then analyzed by western blotting using the anti-Rab3a antibody. The Rab3a levels were decreased in the cells transduced by the shRab3a-encoding lentiviral vector and empty MLV vector (**Figure 3G**), showing that the shRab3a indeed suppressed the endogenous Rab3a expression. The Rab3a amounts were increase in the Rab3a RS-HA-transduced cells. The endogenous CD63 levels were increased by the shRab3a in TE671 cells (**Figures 3G,H**) as well as 293T cells (**Figures 3C,D**), suggesting that the increase of CD63 by the Rab3a silencing was not cell line-specific. The CD63 levels increased by the shRab3a were reduced by the Rab3a RS-HA expression. These results strongly support the conclusion that the Rab3a silencing elevates endogenous CD63 levels.

### Binding between CD63 and Rab3a, and Their Cellular Localizations

**FIGURE 4:**
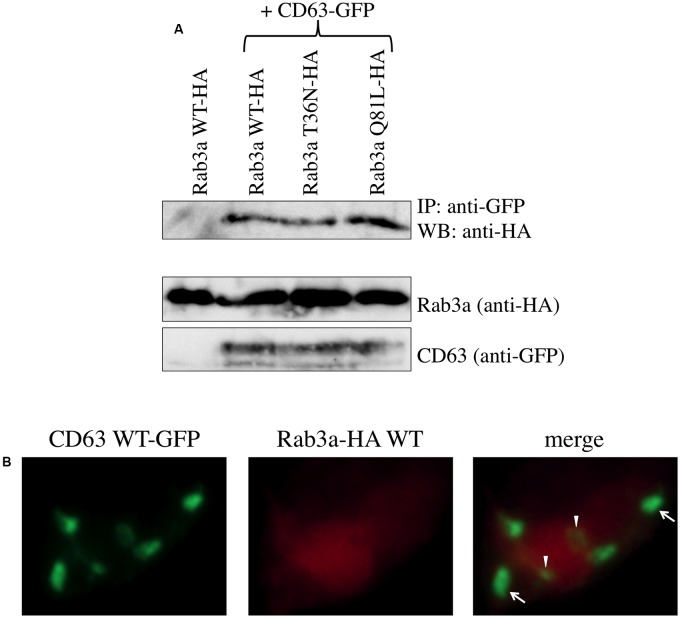
Rab3a binds to CD63. **(A)** 293T cells were transfected using CD63 WT-GFP together with Rab3a WT-HA, T36N-HA, or Q81L-HA. As a control, 293T cells were transfected using Rab3a WT-HA alone. Cell lysates were immunoprecipitated with anti-GFP antibody and the precipitates were analyzed by western blotting using anti-HA antibody. **(B)** 293T cells were transfected using CD63 WT-GFP and Rab3a WT-HA. The transfected cells were permeabilized with methanol. The cells were treated with anti-HA antibody and then with Cy3-conjugated anti-mouse IgG antibody. Green dots indicated by arrows and arrowheads are located in regions where the red signal was weak and strong, respectively.

Rab3a induced the degradation of CD63, so 293T cells co-transfected using the CD63 WT-GFP and Rab3a WT-HA expression plasmids were treated simultaneously with the lysosomal and proteasomal inhibitors to confirm the binding of CD63 and Rab3a. The cell lysates were precipitated using the anti-GFP antibody and the precipitates were then analyzed by western blotting using the anti-HA antibody. The Rab3a WT-HA, T36N-HA, and Q81L-HA proteins were detected in the precipitates (**Figure 4A**). However, when Rab3a WT-HA was expressed alone, Rab3a protein was not detected in the precipitate using the anti-GFP antibody. These results demonstrate that binding occurred between CD63 and Rab3a.

To examine the cellular localization of CD63 and Rab3a, 293T cells were co-transfected using the CD63 WT-GFP and Rab3a WT-HA expression plasmids, and then treated with the lysosomal and proteasomal inhibitors. The transfected cells were permeabilized with methanol, before treating with the anti-HA antibody and then with a Cy3-conjugated anti-mouse IgG antibody. Rab3a protein (red) was distributed widely throughout the cytoplasm, whereas CD63 (green) was detected as several dots in each cell, which represented lysosomes (**Figure 4B**). Most of the green dots were detected in the red area, thereby supporting the binding between CD63 and Rab3a. In particular, the signal strength of the CD63 dots was weak in a region where the red signal was strong (indicated by arrow heads), which indicates that Rab3a induced the degradation of CD63.

**FIGURE 5:**
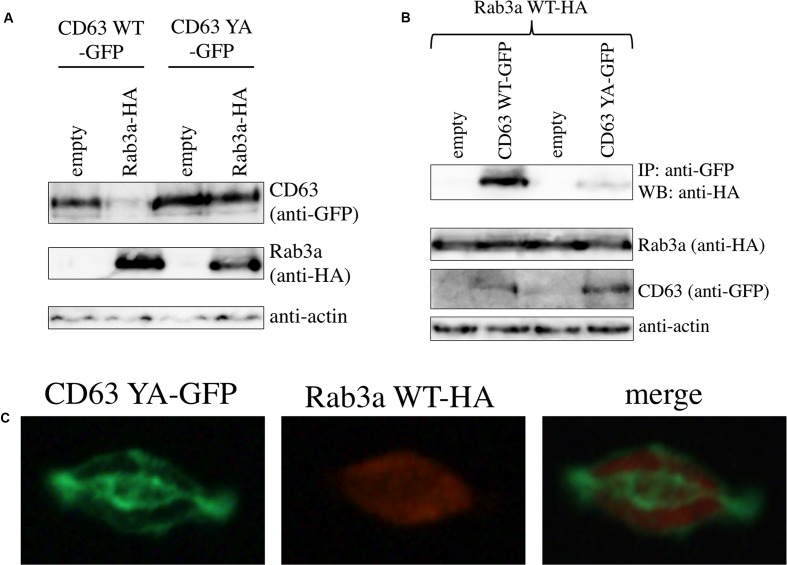
Interaction between CD63 and Rab3a is depend on lysosomal localization of CD63. **(A)** 293T cells were transfected using the CD63 WT-GFP or CD63 YA-GFP expression plasmid together with empty or Rab3a WT-HA expression plasmid. Cell lysates from the transfected cells were analyzed by western blotting with anti-GFP, anti-HA, and anti-actin antibodies. **(B)** 293T cells were transfected using the Rab3a WT-HA expression plasmid together with the empty, CD63 WT-GFP, or CD63 YA-GFP expression plasmid. Cell lysates from the transfected cells were immunoprecipitated with anti-GFP antibody, and the precipitates were analyzed by western blotting with anti-HA antibody. **(C)** 293T cells were transfected using the CD63 YA-GFP and Rab3a WT-HA expression plasmid. The transfected cells were permeabilized with methanol, and treated with anti-HA antibody then with Cy3-conjugated anti-mouse IgG antibody. The cells were observed under a confocal microscope.

To know whether the interaction between Rab3a and CD63 is depend on the lysosomal localization of CD63, a CD63 mutant that does not localize to endosome/lysosome was constructed. The tyrosine residue in the cytoplasmic C-terminal tail of CD63 is the determinant for the endosome/lysosome localization of CD63, and the CD63 mutant that containing an amino acid substitution of the tyrosine residue by alanine (CD63 YA) does not reside in endosome/lysosome (Rous et al., 2002). We constructed an expression plasmid encoding a C-terminally GFP-tagged CD63 YA protein (CD63 YA-GFP). When 293T cells were co-transfected using the expression plasmids of CD63 YA-GFP and Rab3a WT-HA, the levels of CD63 YA-GFP was not changed, although the CD63 WT-GFP level was decreased (**Figure 5A**).

To assess whether CD63 YA mutant binds to Rab3a, 293T cells were transfected using the Rab3a WT-HA expression plasmid together with the pcDNA3.1, CD63 WT-GFP, or CD63 YA-GFP expression plasmid. The Rab3a WT-HA protein amounts co-precipitated with the anti-GFP antibody in the CD63 YA-GFP-containing cell lysates were lower than those in the CD63 WT-GFP-containing cell lysates (**Figure 5B**). This result shows that the tyrosine residue of CD63 is required for the interaction between CD63 and Rab3a.

To examine the cellular localization of CD63 YA mutant, 293T cells were transfected using the expression plasmids encoding CD63 YA-GFP and Rab3a WT-HA. After permeabilization with methanol, the cells were treated with the anti-HA antibody and then with the Cy3-conjugated anti-mouse IgG antibody. Although the CD63 WT-GFP was detected as green dots (**Figure 4B**), pattern of cellular localization of CD63 YA-GFP was completely different from that of CD63 WT-GFP, suggesting that CD63 YA-GFP localized other regions than endosome/lysosome (**Figure 5C**), as already reported (Rous et al., 2002). Taken together, it was suggested that interaction between CD63 and Rab3a is depend on the endosome/lysosome localization of CD63.

### Rab3a Was Not Incorporated into HIV-1 Virions

**FIGURE 6:**
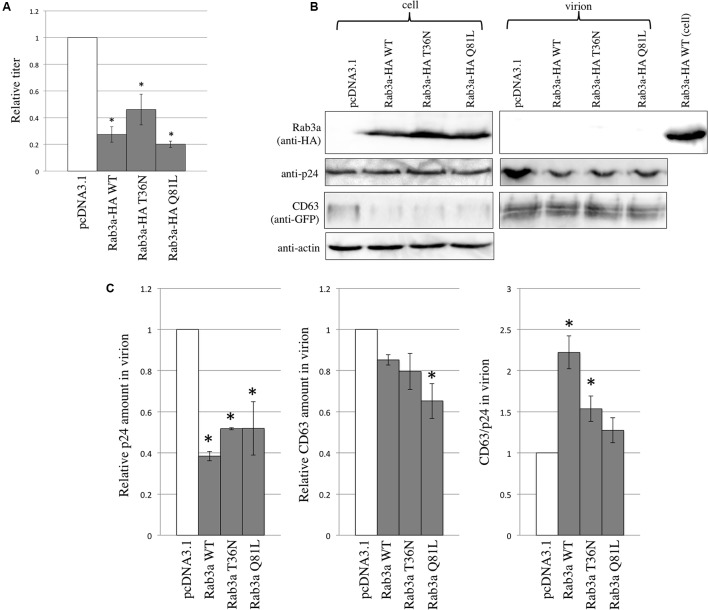
Rab3a overexpression modulated HIV-1 virion formation and Rab3a was not incorporated into HIV-1 virions. **(A)** 293T cells were transfected using the CD63 WT-GFP and VSV-pseudotyped HIV-1 vector construction plasmids together with pcDNA3.1, Rab3a WT-HA, T36N-HA, or Q81L-HA. Culture supernatants from the transfected cells were used to inoculate TE671 cells. Transduction titers from the pcDNA3.1-transfected cells were set to 1 and the relative values ± SD are indicated. Asterisks indicate significant differences compared with the values in the pcDNA3.1-transfected cells. **(B)** Cell lysates and virion pellets were analyzed by western blotting using anti-HA, anti-p24, anti-GFP, and anti-actin antibodies. **(C)** The p24 levels in the cell lysates were normalized against the actin levels. The p24 (left panel) and CD63 (middle panel) levels in the virion pellets were normalized against the normalized p24 levels in the cell lysates. The normalized levels in the pcDNA3.1-transfected cells were set to 1. The normalized CD63 levels were also normalized against the normalized p24 levels in the virion pellets (right panel). Relative values ± SD are indicated. Asterisks indicate significant differences compared with the values in the pcDNA3.1-transfected cells.

To examine the effects of Rab3a on HIV-1 virion formation and to determine whether Rab3a is incorporated into HIV-1 particles, 293T cells were transfected using the CD63 WT-GFP and VSV-pseudotyped HIV-1 vector construction plasmids together with the pcDNA3.1, Rab3a WT-HA, T36N-HA, or Q81L-HA expression plasmid. The transduction titers of the culture supernatants obtained from cells transfected using the Rab3a WT-HA, T36N-HA, or Q81L-HA expression plasmid were slightly lower than those obtained from the pcDNA3.1-transfected cells (**Figure 6A**). Similarly, the HIV-1 p24 levels in virion pellets were decreased slightly by the Rab3a WT-HA, T36N-HA, and Q81L-HA, although the p24 levels in the cell lysates were similar (**Figures 6B,C**). These results indicate that the expression of Rab3a slightly inhibited HIV-1 virion production independently of the Rab3a activation state.

As mentioned above, the amounts of CD63 in the cell lysates were reduced by Rab3a WT-HA, T36N-HA, and Q81L-HA. However, the CD63 levels in the virion pellets were not changed significantly by Rab3a. After normalizing the amounts of CD63 in the virion pellets against the amounts of p24, we found that the CD63 levels were increased by Rab3a WT-HA and T36N-HA. Interestingly, although CD63 interacted with Rab3a and it was detected readily in the virion pellets, Rab3a was not detected in the virion pellets (**Figure 6B**). When the cell lysates and virion pellets were applied to a same gel, levels of p24 and CD63 in the cell lysates and virion pellets were similar, but Rab3a was not detected in the virion pellets (**Supplementary Figure S2**). Thus, Rab3a was not incorporated into HIV-1 particles, which suggests that Rab3a-free CD63 but not Rab3a-bound CD63 was incorporated into the HIV-1 particles.

The experiment described above showed that the expression of exogenous Rab3a inhibited HIV-1 virion formation. To determine whether endogenous Rab3a also inhibits HIV-1 virion formation, the shRab3a-expressing 293T cells were transfected using the VSV-pseudotyped HIV-1 vector construction plasmids. Unexpectedly, the transduction titers of the culture supernatants obtained from the transfected cells were reduced by Rab3a silencing (**Supplementary Figure S3A**). Similarly, the p24 levels in the virion pellets were also decreased (**Supplementary Figures S3B,C**), although the p24 levels in the cell lysates were unchanged. Endogenous CD63 was not detected in the virion pellets prepared from the control and shRab3a-expressing cells. These results indicate that either Rab3a overexpression or silencing suppressed HIV-1 virion production.

## Discussion

In this study, we found that Rab3a binds to CD63 to induce the degradation of CD63. Considering the localization of CD63 in lysosomes, it was reasonable to find that the lysosomal inhibitor hindered the Rab3a-mediated decrease in CD63. However, the protective effect of the proteasomal inhibitor against CD63 degradation was unexpected. Furthermore, polyubiquitinated CD63 and Rab3a were not detected. Thus, Rab3a may activate an unknown cellular factor to induce the lysosomal degradation of CD63 and this factor may be digested in proteasomes.

Although CD63 is involved in HIV-1 virion production, CD63 was not detected in 293T cells that are frequently used as HIV-1 vector-producing cells worldwide. It has been reported that CD81, another member of tetraspanin family, plays an important role in HIV-1 virion production (Grigorov et al., 2009). Thus, other tetraspanin family proteins might be associated with HIV-1 virion production in 293T cells.

We also found that Rab3a-free CD63 was incorporated into HIV-1 particles. CD63 is preferentially incorporated into HIV-1 virions (Chertova et al., 2006) and CD63 interacts with Rab3a. However, Rab3a was not detected in HIV-1 particles. Thus, we consider that Rab3a-free CD63 was incorporated into viral particles and that Rab3a-bound CD63 was degraded. In agreement with this hypothesis, the interaction between Rab3a and CD63 YA mutant that did not localize to lysosomes was weaker than that between Rab3a and CD63 WT.

Rab3a is not involved directly with HIV-1 virion production. Either the overexpression or silencing of Rab3a slightly attenuated HIV-1 virion production. Rab3a was not incorporated into HIV-1 particles. The overexpression of Rab3a reduced the CD63 levels in cells but not in virions. Thus, we suggest that Rab3a affects HIV-1 virion production via a mechanism other than regulating the CD63 levels. Rab3a plays a key role in exocytosis, so changes to exocytosis caused by the overexpression or silencing of Rab3a may modulate HIV-1 virion production.

The exogenous expression of Rab3a reduced the CD63 levels in cells, but the incorporation of CD63 into HIV-1 virions was not attenuated. As mentioned in the Section “Introduction,” it is thought that HIV-1 employs a positive mechanism for preferentially incorporating CD63 into virions. Due to this mechanism, Rab3a might not affect the incorporation of CD63 into HIV-1 particles, even when the CD63 levels are reduced in cells.

The Rab3a T36N-HA and Q81L-HA induced cell death or inhibited cell growth. COS7 cells that stably expressed Rab3a T36N-HA or Q81L-HA were not obtained. Therefore, the constitutively inactive and active mutants induced cell death or suppressed cell proliferation. It has been shown that Rab3a promotes tumor initiation and progression, thereby suggesting that Rab3a is involved with cell growth (Kim et al., 2014). Thus, the exogenous expression of the Rab3a mutants may inhibit cell growth. Alternatively, the modulation of exocytosis by the ectopic expression of the active Rab3a or inactive mutant might induce cytotoxicity because exocytosis is essential for cell viability.

It is generally thought that HIV-1 buds from plasma membrane (Welsch et al., 2007; Votteler and Sundquist, 2013). However, there are many lines of evidence showing that endosomes/lysosomes or lysosome-localized proteins are associated with HIV-1 virion production (Jouve et al., 2007; Molle et al., 2009; Tang et al., 2009; Ha et al., 2012; Liu et al., 2012). In these reports, the lysosomal factors did not affect trafficking of HIV-1 Gag protein. Thus, the endosomes/lysosomes may regulate plasma membrane transport of endosome sorting complex required for transport (ESCORT) that is required for HIV-1 virion formation (Votteler and Sundquist, 2013). To understand this issue, further study is needed.

In summary, this study showed that Rab3a binds to CD63 to induce the degradation of CD63, and that Rab3a-free CD63 is incorporated into HIV-1 particles. Further research is needed to understand the molecular role of CD63 in HIV-1 virion production.

## Materials and Methods

### Cell Lines

Human 293T, human TE671, and African green monkey COS7 cells have been maintained in our laboratory for a long period. They were cultured in Dulbecco’s modified Eagle’s medium with 8% fetal bovine serum and 1% penicillin-streptomycin. To construct Rab3a-silenced cells, 293T cells were transduced using a lentivirus vector encoding an shRNA against Rab3a. The lentivirus vector also expressed a puromycin-resistance gene, so the transduced cells were selected with puromycin. To construct Rab3a-HA WT-expressing COS7 cells, an MLV vector encoding Rab3a-HA WT was inoculated into COS7 cells. The MLV vector also expressed the puromycin-resistance gene, so the inoculated cells were treated with puromycin. The puromycin-resistant cell pools were used in this study.

### Plasmids

Expression plasmids for C-terminally HA-tagged CD63 WT and the TCS mutant were constructed in our previous study (Kubo et al., 2016). The plasmid encoding CD63 tagged with HA and 6× HN (CD63-HA-6× HN) was constructed by ligating the HA-tagged CD63 sequence into the 6× HN tag sequence-containing pET6 × HA plasmid (TaKaRa). The Rab3a sequence-containing plasmid was obtained from the Kazusa DNA Research Institute. To construct the C-terminally HA-tagged Rab3a-expressing plasmid, its protein-coding region was amplified by PCR (TaKaRa). The reverse primer contained the HA tag sequence. The PCR product was cloned into the pTargeT expression plasmid (Promega). The target sequence of shRab3a was GGACAACAUUAAUGUCAAG. The sequence was Rab3a-specific and other transcripts lacked this sequence. The GFP-tagged CD63 expression plasmid was constructed in our previous study (Izumida et al., 2016). The mutants of CD63 and Rab3a were constructed by the standard PCR-mediated site-directed mutagenesis (TaKaRa), and their nucleotide sequences were confirmed by sequencing (Applied Biosystems). The HIV-1 Gag-pol expression plasmid (R8.91) was kindly provided by Dr D. Trono (Naldini et al., 1996). The expression plasmids of the LacZ-encoding HIV-1 vector genome and VSV-G were provided by Dr L. J. Chang (Chang et al., 1999; Iwakuma et al., 1999).

### Isolation of CD63-Binding Proteins

COS7 cells were transfected using the CD63-HA-6× HN expression plasmid. Cell lysates were prepared from the cells at 2 days after transfection. The cell lysates were applied to Ni columns (TaKaRa). The CD63-binding proteins were eluted using imidazole-containing buffer and fractionated to obtain 0.5 ml of each. The CD63-containing fractions were subjected to SDS-PAGE and silver staining. A protein band that was detected in CD63 WT but not in the TCS mutant was picked from the SDS-PAGE gel. The gel band was destained according to the manufacturer’s instructions (Silver Stain MS kit, Wako, Japan). The protein in gel was treated with trypsin and eluted from the gel according to the manufacturer’s instructions (Bruker Daltonics, Germany). The trypsin-digested sample was mixed with the same volume of 0.7 mg/ml a-cyano-4-hydroxycinnamic acid (HCCA; Bruker Daltonics, Germany) as a matrix. The HCCA-mixed sample was analyzed by Ultraflex III matrix assisted laser desorption/ionization–time of flight mass spectrometry (Bruker Daltonics, Germany) using the associated software [flexcontrol (ver. 3.3) and flexanalysis (ver. 3.3)] and the *m/z* peaks were obtained. The *m/z* signals were analyzed using biotools (ver. 3.2; Bruker Daltonics, Germany) and matrix server (Matrix Science, United States) to identify the protein in a public database (Swiss-Prot).

### Construction of HIV-1 Vector

COS7 or 293T cells were transfected with the expression plasmids of HIV-1 Gag-pol, LacZ-encoding HIV-1 vector genome, and VSV-G using Fugene transfection reagent (Promega). The culture media were replaced with fresh media at 24 h after transfection and the cells were cultured for 24 h more. The culture media were used to inoculate the target TE671 cells. The inoculated cells were stained with X-Gal at 2 days after inoculation and blue cells were counted to estimate the transduction titers.

### Isolation of Virion-Containing Fraction

Culture supernatants were centrifuged at 1,000 rpm for 5 min to remove cells and cell debris. The supernatants were then centrifuged at 14,000 rpm for 4 h through 20% sucrose. The pellets were suspended in PBS and analyzed by western blotting.

### Western Blotting

Cell lysates or virion pellets were subjected to SDS-PAGE (Bio-Rad) and the proteins were transferred to PVDF membranes (Millipore). The membranes were treated with mouse anti-GFP (Nacalai Tesque), anti-HA (Covance), or anti-CD63 (Santa Cruz Biotechnology) antibody and then with HRP-conjugated anti-mouse IgG antibody (Bio-Rad). The antibody-bound proteins were visualized using the ECL reagent (Bio-Rad).

### Immunoprecipitation

To assess the binding between CD63-GFP and Rab3a-HA, mouse anti-GFP antibody (Nacalai Tesque) was added to the cell lysates and incubated at 4°C for 4 h. Anti-mouse IgG antibody-agarose beads (Sigma-Aldrich) were added to the sample and incubated for 4 h. The beads were washed and proteins were eluted using SDS-containing sample buffer. The elutes were subjected to SDS-PAGE and western blotting with anti-HA antibody and then with the anti-native mouse IgG antibody (GeneTex).

To detect endogenous Rab3a, rabbit anti-Rab3a antibody was added to the cell lysates. Elutes from the anti-rabbit IgG antibody-beads were subjected to SDS-PAGE and western blotting with anti-Rab3a antibody, and then with the anti-native rabbit IgG antibody (GeneTex).

### Statistical Analysis

Differences between two sets of data were analyzed using the Student’s *t*-test and differences were considered significant at *P* < 0.05.

## Author Contributions

YK and MI performed the experiments of this study. HM carried out the mass spectrometry. YK, MI, KK, HH, and TM analyzed the data. YK and HH wrote the manuscript.

## Conflict of Interest Statement

The authors declare that the research was conducted in the absence of any commercial or financial relationships that could be construed as a potential conflict of interest.

## Supplementary Material

FIGURE S1 CD63-binding proteins were isolated. **(A)** COS7 cells were transfected using CD63 WT-HA-6× HN or CD63 TCS-HA-6× HN. Cell lysates were applied to Ni columns. CD63-binding proteins were eluted using an imidazole-containing buffer and fractionated to obtain 0.5 ml of each. The fractions were analyzed by western blotting using anti-HA antibody. **(B)** The CD63-containing fractions were subjected to SDS-PAGE and silver staining. The additional band detected in CD63 WT but not in CD63 TCS is indicated by an arrow.

FIGURE S2 Rab3a was not incorporated into HIV-1 particles. 293T cells were transfected using the CD63 WT-GFP and HIV-1 vector construction plasmids together with pcDNA3.1 or Rab3a WT-HA expression plasmid. Cell lysates and virion pellets prepared from the transfected cells were applied to a same gel, and western blotting was performed.

FIGURE S3 Rab3a silencing modulated HIV-1 virion formation. **(A)** 293T cells transduced using the empty or shRab3a-expressing lentivirus vector were transfected with the VSV-pseudotyped HIV-1 vector construction plasmids. Culture supernatants from the transfected cells were used to inoculate TE671 cells. Transduction titers from the empty vector-transduced cells were set to 1 and the relative values ± SD are indicated. Asterisk indicates significant differences compared with the values in the empty vector-transduced cells. **(B)** Cell lysates and virion pellets prepared from the transfected cells were analyzed by western blotting using anti-p24 and anti-actin antibodies. **(C)** The p24 levels in the cell lysates were normalized against the actin levels. The p24 levels in the virion pellets were normalized against the normalized p24 levels in the cell lysates. The p24 levels in the empty vector-transduced cells were set to 1 and the relative values are indicated. Asterisk indicates significant differences compared with the values in the empty vector-transduced cells.
