# Supplementary figures and images for: Rab3a-Bound CD63 Is Degraded and Rab3a-Free CD63 Is Incorporated into HIV-1 Particles
Source: Front Microbiol. 2017 Aug 29;8:1653. doi: 10.3389/fmicb.2017.01653 (PMC5581869; doi:10.3389/fmicb.2017.01653)

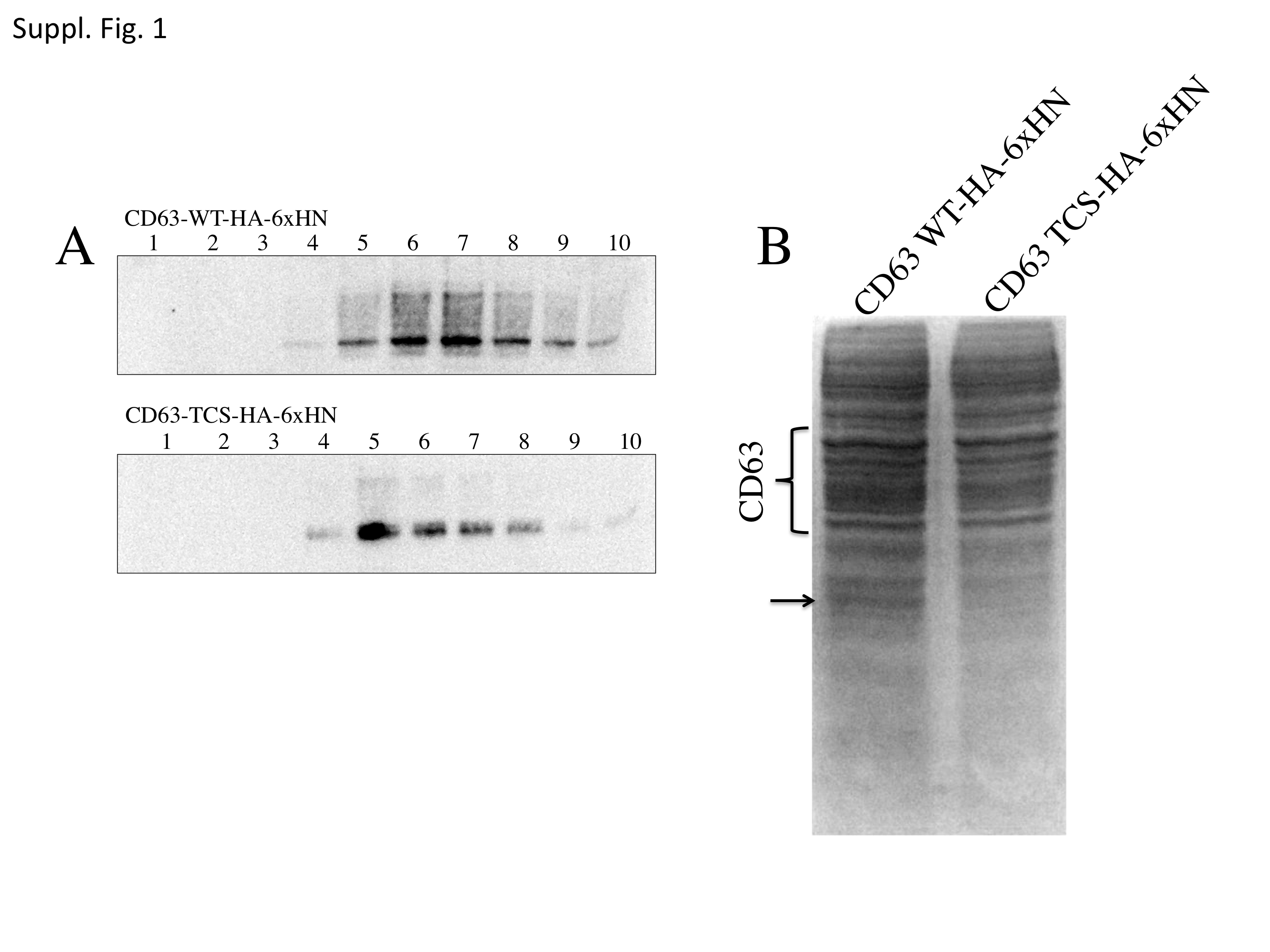

Supplement: FIGURE S1 — CD63-binding proteins were isolated. (A) COS7 cells were transfected using CD63 WT-HA-6× HN or CD63 TCS-HA-6× HN. Cell lysates were applied to Ni columns. CD63-binding proteins were eluted using an imidazole-containing buffer and fractionated to obtain 0.5 ml of each. The fractions were analyzed by western blotting using anti-HA antibody. (B) The CD63-containing fractions were subjected to SDS-PAGE and silver staining. The additional band detected in CD63 WT but not in CD63 TCS is indicated by an arrow. [file Image_1.TIF]

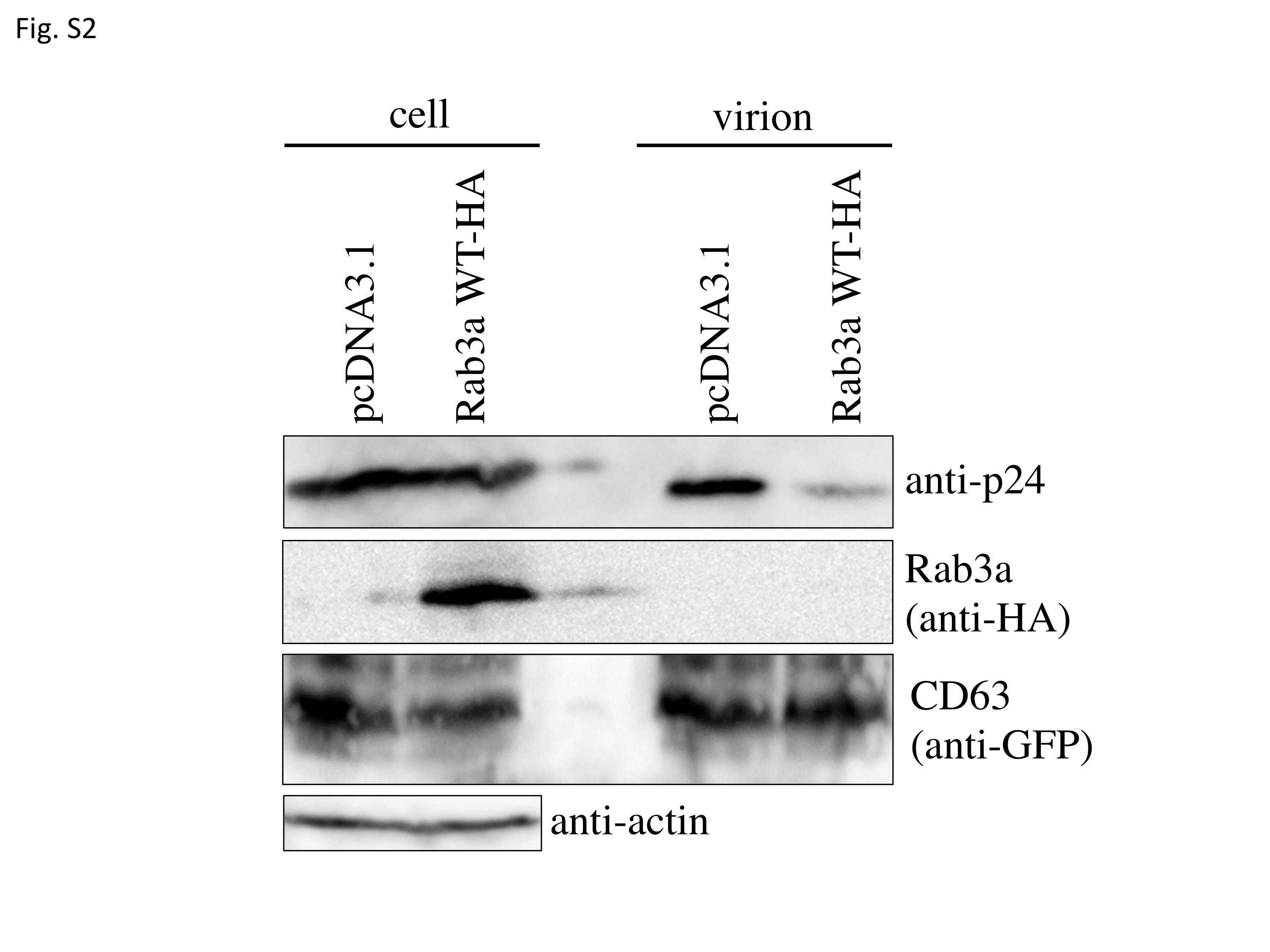

Supplement: FIGURE S2 — Rab3a was not incorporated into HIV-1 particles. 293T cells were transfected using the CD63 WT-GFP and HIV-1 vector construction plasmids together with pcDNA3.1 or Rab3a WT-HA expression plasmid. Cell lysates and virion pellets prepared from the transfected cells were applied to a same gel, and western blotting was performed. [file Image_2.TIF]

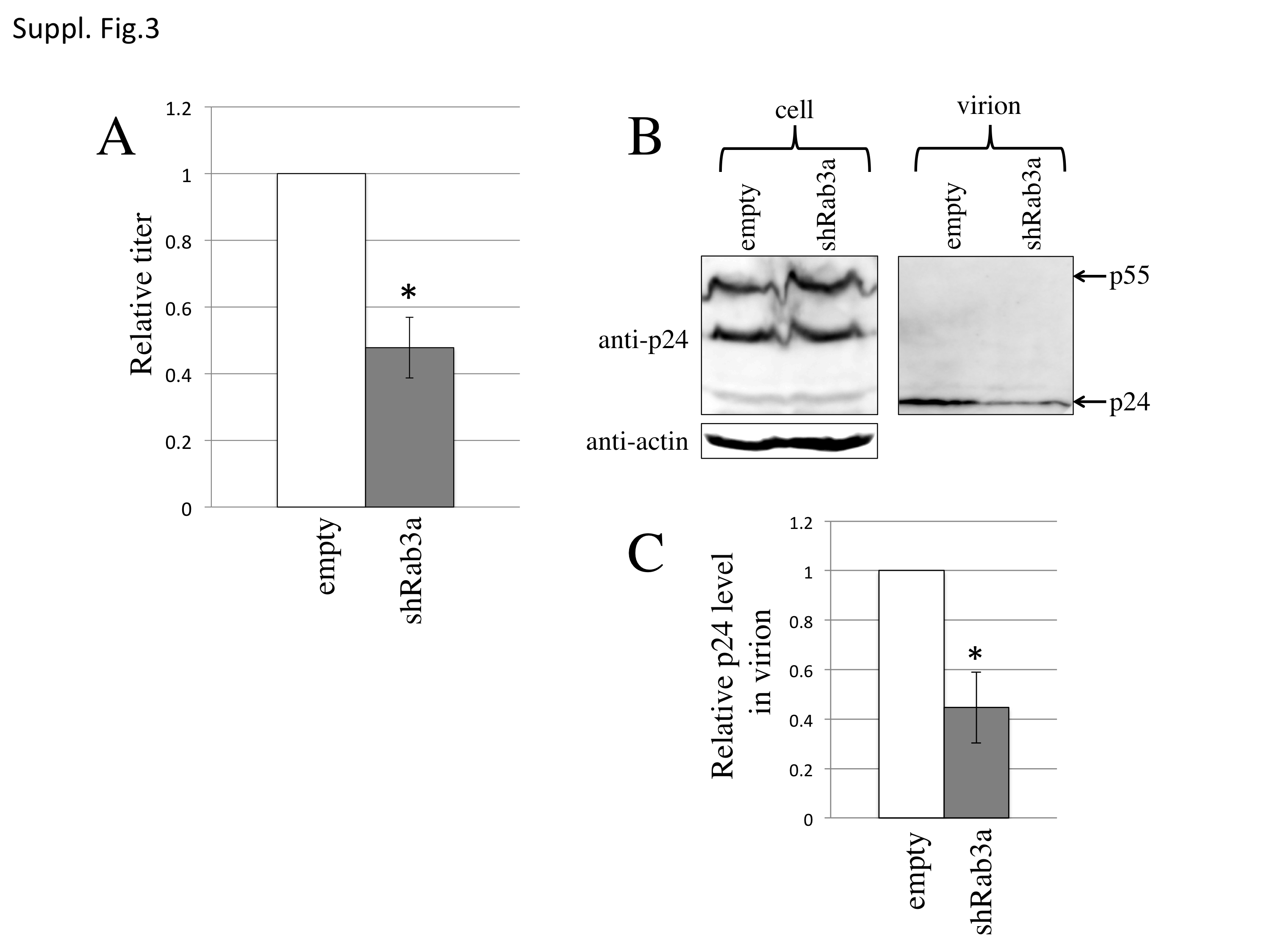

Supplement: FIGURE S3 — Rab3a silencing modulated HIV-1 virion formation. (A) 293T cells transduced using the empty or shRab3a-expressing lentivirus vector were transfected with the VSV-pseudotyped HIV-1 vector construction plasmids. Culture supernatants from the transfected cells were used to inoculate TE671 cells. Transduction titers from the empty vector-transduced cells were set to 1 and the relative values ± SD are indicated. Asterisk indicates significant differences compared with the values in the empty vector-transduced cells. (B) Cell lysates and virion pellets prepared from the transfected cells were analyzed by western blotting using anti-p24 and anti-actin antibodies. (C) The p24 levels in the cell lysates were normalized against the actin levels. The p24 levels in the virion pellets were normalized against the normalized p24 levels in the cell lysates. The p24 levels in the empty vector-transduced cells were set to 1 and the relative values are indicated. Asterisk indicates significant differences compared with the values in the empty vector-transduced cells. [file Image_3.TIF]
